# The influence of social class of origin on labor market entry and the mediating role of education in Italy

**DOI:** 10.3389/fsoc.2025.1585459

**Published:** 2025-06-25

**Authors:** Davide Bussi, Carlotta Piazzoni, Marta G. Pancheva, Mario Lucchini

**Affiliations:** ^1^Department of Sociology and Social Research, University of Milano-Bicocca, Milan, Italy; ^2^Department of Social and Political Sciences, Economics and Management, Sophia University Institute, Figline and Incisa Valdarno, Italy

**Keywords:** social origin, education mediation, labor market entry, socio-economic inequalities, event history analysis, Italian Lives (ITA.LI)

## Abstract

**Introduction:**

Access to the labor market is influenced by various socio-economic factors, including social class and education. In Italy, these elements play a crucial role in determining employment opportunities and career trajectories. This study aims to analyze how social origin influences transition to the first job across different birth cohorts, gender groups, and macro-region of residence while also assessing the mediating role of education.

**Methods:**

Using Event History Analysis, we estimate labor market entry timing via survival models and discrete-time logistic regression, accounting for social background effects. We classify social origin using the European Socio-economic Classification scale based on the parental occupation. The analysis, conducted separately by gender, controls for birth cohort, education, parenthood, and area of residence. Also, we employ the KHB decomposition, which enables us to quantify the extent to which education mediates the influence of social background on labor market entry.

**Results:**

Individuals from lower social backgrounds enter the labor market earlier, while those from higher-status families tend to delay entry, likely due to extended education and greater financial support. Educational attainment mediates the relationship between social origin and labor market entry, as individuals from higher-status backgrounds tend to delay entry due to prolonged education. However, education does not fully eliminate class-based disparities—controlling for educational attainment amplifies rather than erases the effect of social origin, indicating that other mechanisms still play a role. Social class disparities in labor market entry remain largely stable across cohorts, with only minimal convergence among men and no significant change among women. For men, class effects remain stable across macro-regions, while for women they are stronger in northern Italy.

**Discussion:**

Our findings confirm that social origin remains a significant determinant of labor market entry in Italy, despite changes in education and labor market structures over time. While increased access to education has contributed to greater opportunities, it has not entirely eliminated class-based disparities in employment transitions.

## 1 Introduction

Transitions to adulthood—such as completing education, entering the labor market, forming a family, and having a child—have been increasingly postponed in contemporary societies. This shift reflects broader social and economic changes that have redefined traditional pathways to adulthood, making them more varied and prolonged over time (Billari and Liefbroer, 2010). Among these major transitions to adulthood, entering the labor market holds particular significance. The centrality of work in any society is almost self-evident, as it represents not only a source of income but also a crucial element for personal and social wellbeing. After completing formal education, seeking employment becomes a prerequisite for achieving financial and family independence. However, the timing and manner in which individuals enter the labor market vary significantly, influencing social mobility and contributing to the intergenerational reproduction of inequality (Blossfeld and Rohwer, 1997).

Delaying entry into the labor market can offer clear advantages for individuals who extend their education to acquire professional qualifications, enhancing career prospects, although it may result in lower lifetime earnings and delayed transitions to marriage and parenthood.

In line with theories on human capital and occupational mobility, a delay in labor market entry is generally associated with better outcomes for individual and professional wellbeing. According to Becker's (1976) human capital theory, spending more time on education allows for greater skill acquisition, increasing a worker's value in the labor market and improving career and income opportunities over the long term.

Conversely, early labor market entry often means lower specialization and qualification, risking the individual being trapped in low-wage, insecure positions with limited chances for advancement (Blossfeld and Mills, 2005). This is particularly evident in segmented labor markets (Doeringer and Piore, 1971), where early entrants are often confined to the secondary sector with precarious jobs, while the primary sector offers more stability and opportunities for growth.

From a wellbeing perspective, early labor market entry is linked to higher stress, lower economic security, and challenges in balancing work and personal life (Schoon and Silbereisen, 2009). Additionally, early and unstable career paths can have cumulative negative effects, limiting earning potential and increasing the risk of economic insecurity in adulthood (DiPrete and Eirich, 2006).

Thus, delaying labor market entry, when combined with investments in education and training, can be a beneficial strategy for both occupational stability and personal wellbeing, leading to greater fulfillment in both professional and personal life.

Several factors shape the age at which individuals secure their first job. In recent decades, all Western countries have experienced a rising age of labor market entry, largely driven by the increasing time spent in education (Pisati, 2002; Lucchini and Schizzerotto, 2001). However, education is not the only factor at play. Among them, this study focuses on the role of social class of origin, which acts as a distal determinant that remains relatively underexplored in the literature. Additionally, we examine the mediating role of educational attainment in this relationship.

Prior research has established that social background significantly shapes educational trajectories (Bourdieu and Passeron, 1977; Erikson and Jonsson, 1996), which, in turn, affect the timing and nature of labor market entry (Breen and Goldthorpe, 1997). However, the extent to which social class directly accelerates or delays workforce entry—beyond its influence on educational pathways—remains an open question requiring further investigation.

In contexts like Italy, where overall labor market mobility is low and the connection between education and employment remains weak, class-based disparities in the timing of labor market entry are particularly pronounced. Youth from more advantaged families are often able to delay entry while pursuing higher education or waiting for better job matches, supported by financial and social resources. In contrast, those from disadvantaged backgrounds tend to enter earlier, often out of necessity, and into less secure positions—thereby reinforcing social inequalities from the outset of their careers (Lucchini and Schizzerotto, 2001).

By applying life course research and event history analysis techniques to retrospective panel data from ITA.LI, we explore the experiences of young Italians over the past 60 years. This study specifically examines how social origin influences transition to the first job across different birth cohorts and gender groups while also assessing the mediating role of education. Our objective is to enhance understanding of whether structural inequalities persist or evolve across generations in the Italian context.

The paper is structured into five sections. Following this introduction, Section 2 provides the contextual background on labor market dynamics in Italy. Section 3 outlines the theoretical framework and presents the research hypotheses. Section 4 details the data, variables, and statistical models used in the analysis, while Section 5 presents the empirical results. Finally, Section 6 offers concluding remarks.

## 2 Contextual background

A comprehensive analysis of the timing of entry into the labor market requires an in-depth examination of the key historical and institutional phases that have shaped Italy's economic development, as well as the evolution of its labor and education policies. These factors are crucial for understanding how social origin influences labor market trajectories, as they establish the broader framework within which individuals navigate their transitions to adulthood.

Italy, like other Western countries, transitioned from a predominantly agrarian economy to an industrial one, initially shaped by Taylorist-Fordist principles that emphasized mass production and high productivity. However, this industrial model was later supplanted by the rise of a service-based economy characterized by labor market flexibility and widespread precarious employment (Mingione and Pugliese, 2002). This shift set the stage for broader changes in both the labor market and in the timing of labor market entry for young adults.

From 1950 to 1973, Italy experienced remarkable economic growth, with per capita GDP rising at an annual rate of over 5%. This period, known as the “Glorious Thirty,” allowed Italy to catch up with leading industrial nations in terms of productivity, output, and welfare, aided by innovations, structural reforms, and the consolidation of the welfare state.

Several reforms contributed to Italy's economic transformation, including key educational changes. In 1962, the Italian government introduced the school reform that made secondary education (middle school) compulsory for all students, significantly increasing access to education for young people across the country (Saraceno, 1963). This reform was instrumental in expanding educational opportunities, reducing illiteracy rates, and promoting social mobility, thus laying the groundwork for a more equitable labor market.

In 1969, the university reform further democratized access to higher education by removing the entrance exams for most universities and extending university access to a broader segment of the population, particularly for working-class students (Pugliese and Rebeggiani, 1997). This move was vital for reducing educational inequalities and enabling greater upward mobility through higher education. Together, these reforms transformed the educational landscape in Italy, providing more young people with the skills and credentials needed to enter the labor market, even as the economy continued to evolve.

However, by the 1970s, the economic model based on these advantages had begun to slow. The need for more profound reforms to support a post-Fordist economy, based on knowledge, technological innovation, and modernized institutions, was not fully addressed (Bastasin and Toniolo, 2020). In response to the crisis of Fordist capitalism, Italy saw a shift away from large, hierarchical firms and toward small and medium-sized enterprises (SMEs) with a focus on specialized, high-quality products (Piore and Sabel, 1984). This transformation was accompanied by significant changes to labor market regulations, starting with the 1970 Workers' Statute, which aimed to protect workers' rights but also contributed to labor market rigidities. As Italy moved into the 1980s and 1990s, a series of labor reforms introduced more flexible employment contracts, such as part-time and fixed-term contracts, in response to increasing demands for labor market flexibility. However, these reforms disproportionately affected young workers, leading to a greater reliance on temporary and precarious employment (Gualmini and Rizza, 2013).

In response to the crisis of Fordist capitalism, Italy saw a shift away from large, hierarchical firms and toward small and medium-sized enterprises with a focus on specialized, high-quality products (Piore and Sabel, 1984). This transformation was accompanied by significant changes to labor market regulations, starting with the 1970 Workers' Statute, which aimed to protect workers' rights but also contributed to labor market rigidities. As Italy moved into the 1980s and 1990s, a series of labor reforms introduced more flexible employment contracts, such as part-time and fixed-term contracts, in response to increasing demands for labor market flexibility. However, these reforms disproportionately affected young workers, leading to a greater reliance on temporary and precarious employment (Gualmini and Rizza, 2013).

These transformations also had gendered implications. Since the postwar period, male and female labor force participation in Italy has evolved along markedly different trajectories. While male employment remained high and relatively stable, women's participation experienced a bifurcated pattern: it declined between the 1950s and the mid-1970s, followed by a steady increase from the late 1970s to the present (Mingione and Pugliese, 2002; Fullin and Reyneri, 2015). This upward trend has been most pronounced among highly educated women (Bozzon, 2008). In fact, women in Italy are now more educated than men, with 68% of women aged 25–64 holding at least an upper secondary qualification compared to 62.9% of men, and 24.9% holding a tertiary degree vs. 18.3% of men—yet this educational advantage has not translated into equal labor market outcomes (ISTAT, 2025). Women's entry into the labor market continues to be shaped by persistent structural and cultural barriers. Italy's familialistic welfare model has traditionally allocated caregiving responsibilities to the private sphere, with limited public investment in childcare and eldercare services (Esping-Andersen, 1990; Saraceno, 2003). These constraints have contributed to fragmented and discontinuous female employment trajectories, particularly among women with low educational attainment or from Southern regions (Bozzon, 2008; Mencarini and Solera, 2011).

Despite recent gains, the female employment rate in Italy remains significantly lower than in Northern European countries, and the share of women in part-time employment is disproportionately high—a pattern shaped by enduring social norms assigning caregiving roles predominantly to women and by the underdevelopment of both public and private care infrastructures (Naldini and Saraceno, 2022). Labor market deregulation in the early 2000s, particularly through the Biagi Law (Italy, 2003), expanded flexible contract types, fostering women's and youth participation but increasing precarity and delaying entry into stable employment (Fullin and Reyneri, 2015). Notably, while women's overall entry has risen only modestly, a substantial shift has occurred in their sustained participation: exits from the labor market due to family reasons declined from 42% in the 1950s to 10% in the 2000s, signaling stronger work attachment—especially among higher-educated women able to secure more qualified, better-paid jobs (Fullin and Reyneri, 2015).

These gendered and institutional dynamics have unfolded alongside—and often been exacerbated by—broader global and regional economic shifts. Italy's economic trajectory was profoundly affected by the 2008 global financial crisis, which led to a steep decline in youth employment, particularly between 2008 and 2013. The employment rate for young people (aged 15–34) fell by over 10 percentage points, exacerbating pre-existing labor market challenges. In recent decades, Italy's position among the world's most advanced economies has deteriorated, with rising debt, political instability, and uneven regional development (Bastasin and Toniolo, 2020).

A significant aspect of the Italian labor market is the persistent territorial divide between the North and South, which dates back to the country's unification. While youth unemployment and gender disparities in labor market participation are relatively moderate in the North, these issues are far more acute in the South, where a lack of regular employment opportunities and patriarchal cultural models continue to reinforce gender inequality (Reyneri, 1997). In this context, a deeper understanding of the timing of labor market entry must also account for the different regional dynamics and how these disparities affect young people's access to the labor market.

In Italy, prolonged residence within the family of origin and the delay in entering the workforce have become pressing social issues, with significant implications for both the fertility rate and the pension system. Various scholars have sought to identify the causes of this “syndrome of delay” by examining an array of individual and institutional factors. They highlight weak connections between the educational system and the job market, shifts in social norms and individual expectations, and a familistic and minimally protective welfare system that offers little support for young people (Hannan et al., 1996; Galland, 1995; Sabbadini, 1999; Dalla Zuanna, 2001; Barbagli et al., 2003; Livi Bacci, 2008).

This historical and institutional context provides an essential backdrop for analyzing the timing of labor market entry. While existing research suggests that young people's trajectories vary by social background and regional opportunity structures, the extent to which Italy's economic transformations have reshaped these class-based patterns remains an open empirical question. This study aims to investigate how these dynamics have evolved and whether traditional inequalities have persisted or changed over time.

## 3 Theoretical background and research hypotheses

Sociological literature has widely debated whether structural transformations in advanced societies have led to greater individualization of life trajectories—a process described as “de-standardization” (Beck, 1999; Giddens, 1994; Bauman, 2000; Bruckner and Mayer, 2005). From a demographic perspective, the “Second Demographic Transition” (Lesthaeghe and Van de Kaa, 1986) similarly highlights how transitions to adulthood, including labor market entry, have become less bound to traditional social structures. Within this framework, the influence of social origin is thought to have weakened over time, as institutional reforms such as educational expansion, labor regulation, and welfare development have broadened access to resources and reduced inequality (Clark and Lipset, 1991; Esping-Andersen, 1990).

Indeed, Italy's education reforms—particularly the 1962 school reform and the 1969 university reform—marked important turning points in the massification of education and the democratization of access (Saraceno, 1963; Pugliese and Rebeggiani, 1997). These reforms are often viewed as mechanisms for reducing the influence of family background on life trajectories. Welfare expansion and labor regulation further contributed to this shift by improving equality of opportunity, particularly in Northern and Central Italy (Korpi, 2000).

However, other scholars challenge the assumption that social origin has lost its relevance. Despite structural changes, empirical research points to the persistence of class-based inequality, especially in societies where educational and labor market hierarchies remain entrenched (Goldthorpe, 2000; Piketty, 2014). The persistence of wealth concentration, the growing importance of cultural capital, and the reproduction of elite advantages through social networks suggest that while the mechanisms of inequality may have evolved, social origin still plays a significant role in shaping individual outcomes. In Italy, this is particularly evident in the phenomenon of delayed labor market entry, or the so-called “syndrome of delay,” linked to weak school-to-work transitions and a familistic welfare model offering limited support to young people (Hannan et al., 1996; Galland, 1995; Sabbadini, 1999; Dalla Zuanna, 2001; Barbagli et al., 2003; Livi Bacci, 2008).

The “cumulative disadvantage theory” (Crystal and Shea, 1990; DiPrete and Eirich, 2006) further highlights how early-life disadvantages tend to compound over time, reinforcing cycles of inequality. Individuals who experience constrained opportunities in education and employment early on are more likely to face economic instability and precarious life conditions later in adulthood. Socioeconomic inequalities, once established, persist and intensify across the life course, leading to widening disparities in wellbeing, career stability, and overall life satisfaction (Liefbroer and Zoutewelle-Terovan, 2021).

Empirical research on this topic has produced mixed findings. Some studies highlight a strong and persistent effect of family background on labor market entry (Elzinga and Liefbroer, 2007; Ravanera et al., 2006; Fraboni and Sabbadini, 2014), while others suggest that once educational attainment is accounted for, the role of social origin becomes negligible (Schizzerotto, 2002; Fullin and Reyneri, 2015). A recent comparative analysis across Europe further demonstrates that socioeconomic background significantly shapes the timing of labor market entry, with higher-SES individuals typically entering employment later due to prolonged educational trajectories (Ferraretto and Vitali, 2024). Based on this evidence, we formulate our first hypothesis:

H1: *Individuals from lower social class backgrounds enter the labor market at a younger age compared to those from higher social class backgrounds*.

To understand how these mechanisms operate, we draw on a resource-based perspective (Bourdieu, 1984; Granovetter, 1973) and a life course framework (Elder, 1985; Settersten and Hagestad, 1996), both of which emphasize the importance of early-life conditions and accumulated resources in shaping key transitions in adulthood. Economic and cultural capital, along with social and symbolic resources, influence young people's decisions around education, job seeking, and family formation (Billari et al., 2019). Higher-socioeconomic status (SES) families provide financial security, educational support, and cultural capital, enabling their children to pursue extended education and career development. Conversely, young adults from lower-SES families often face financial constraints and structural barriers that accelerate transitions such as entering the job market, leaving home, or becoming a parent (Furstenberg, 2010; McLanahan, 2004). According to Billari et al. (2019), three key mechanisms mediate the relationship between parental socioeconomic background and young adults' demographic decisions: stratified socialization, stratified agency, and stratified opportunity.

Among these mechanisms, education stands out as a central pathway through which social origin shapes labor market outcomes, serving as both a resource and a filter in the transition to employment. In fact, education is often viewed as the most critical mediating channel between social origin and labor market outcomes. While it is often conceptualized as a tool for promoting merit-based mobility (Becker, 1976), it may also reinforce existing inequalities if access to quality education is uneven or if credentials are insufficient to overcome class-based disparities in hiring practices (Bourdieu, 1984; Schizzerotto, 2002). Based on this, we hypothesize:

H2: *Educational attainment mediates the relationship between social origin and labor market entry but does not fully eliminate class-based disparities*.

This mediating role of education may be especially salient for women. Women's entry into the labor market is shaped not only by class-based inequalities but also by deeply embedded gendered norms and institutional constraints. Research shows that even highly educated women face persistent barriers to stable employment due to occupational segregation, caregiving responsibilities, and employer discrimination (Blau and Kahn, 2017; ISTAT, 2025). Italy's welfare model remains strongly familialistic, with weak state support for early childcare and long-term care, especially in the South (Saraceno, 2003; Naldini and Saraceno, 2022). These conditions reinforce the gendered division of labor, particularly penalizing mothers of young children and women from lower socioeconomic backgrounds. Employer stereotyping also plays a significant role, as women—especially those who are young and of childbearing age—are often perceived as less reliable or more costly employees, regardless of their qualifications (Bozzon, 2008; Naldini and Saraceno, 2022). Moreover, women from lower social classes tend to enter the labor market earlier, often into precarious and segmented forms of work, due to both economic necessity and limited access to supportive networks or quality education (ISTAT, 2025; Mencarini and Solera, 2011). In contrast, highly educated women may delay labor market entry due to prolonged education but still face a slower transition into stable employment. These mechanisms suggest that the relationship between social origin and labor market entry is not only classed but also gendered, and that education plays a differentiated mediating role for men and women.

H3: *The effect of social origin on labor market entry is gendered, and the mediating role of education is stronger for women*.

Finally, the impact of social origin is also likely to vary across birth cohorts. Structural changes in the labor market—including rising job precarity, deregulation, and economic crises—may have weakened the role of class by affecting broader segments of the youth population (Reyneri, 2005; Ferraretto and Vitali, 2024). While older cohorts may have benefited from more stable transitions, younger generations face prolonged school-to-work pathways and more fragmented career starts. This motivates our final hypothesis:

H4: *The influence of social origin on labor market entry has weakened across birth cohorts due to educational expansion, while labor market deregulation and economic crises have increased precarity across all social groups, potentially attenuating class-based disparities*.

These hypotheses guide the empirical analysis that follows, which aims to assess the extent to which social origin continues to shape the transition to work in Italy, and whether education—interacting with gender and historical context—can serve as a pathway to equalizing life chances.

## 4 Data and analytical strategies

### 4.1 Data and variables

For the purposes of our analysis we used retrospective longitudinal data on working careers taken from the Italian Lives survey (ITA.LI) (Lucchini et al., 2023). The first wave of the ITA.LI survey, launched in 2019 by the IASSC (Institute for Advanced Study of Social Change) at the Department of Sociology and Social Research of the University of Milano-Bicocca collected, between the end of 2019 and the first months of 2021, the information necessary to reconstruct the life courses of the individuals (4,900 households). Participants were selected from over 250 Italian municipalities through a three-stage probabilistic sampling design developed with ISTAT (Pisati, 2023). The survey reconstructs individuals' trajectories from birth to the time of the interview, covering school career, family life, residential mobility, and employment history. The ITA.LI survey represents the most comprehensive and detailed longitudinal dataset currently available collecting information on individuals living in Italy, offering unparalleled insight into the interplay between life course dimensions and social stratification. Specifically, the database used to study the entry into the job market consists of ~6,000 subjects, for whom it was possible to reconstruct the entire career on a monthly basis, from the first episode of employment or unemployment up to the date of the interview. Specifically, for the purpose of this study we will focus on the first entry into the job market. This is defined as the individual's first substantial work experience, explicitly excluding minor or informal activities. During data collection, respondents were instructed not to include occasional or marginal jobs—such as small tasks undertaken to cover personal expenses and to report only those employment episodes that marked a genuine transition into the labor force. As such, the variable captures meaningful labor market entry, providing a more robust indicator of integration into employment and allowing us to assess class- and gender-based differences in labor market entry.

We classify social origin using the European Socio-economic Classification scale, based on the parental occupation when the respondent was 14 years old, divided into five categories: 1-Employer, Managers and Professionals; 2-Higher and Mid White Collars; 3-Self-employed; 4-Lower Technical and Skilled Manual Workers; 5-Lower Sales and Routine Manual. Specifically, we used the highest class between mothers and fathers, according to the dominance approach (Erikson, 1984).

We also stratify by gender and control for birth cohort (1926–1945 Silent Generation; 1946–1955 Baby Boomers 1; 1956–1965 Baby Boomers 2; 1966–1980 Generation X; 1981–1995 Generation Y; 1996–2004 Generation Z).

Moreover, we have used a series of time-varying covariates, namely: area of residence (North-West, North-East, Centre, South, Islands, Abroad); highest educational degree obtained (Primary, Lower Secondary, Upper Secondary, Tertiary, Post-Tertiary); entry into parenthood; and current enrollment in education.

### 4.2 Analytical strategies

To analyze labor market entry, we employ Event History Analysis (EHA) techniques, which allow us to model the transition from labor market inactivity to employment while accounting for the timing of this event (Blossfeld and Rohwer, 1997). Specifically, we estimate survival functions over time and apply discrete-time survival models to a person-period dataset. This approach enables us to assess the hazard of transitioning into employment within predefined time intervals, examining how this risk varies by social background while controlling for other relevant covariates.

Given the interdependent nature of life course trajectories, we also examine the interplay between labor market entry and other key life events, such as educational attainment, family formation, and geographical mobility. We recognize their reciprocal influence on career transitions and assess their potential mediating and/or moderating role in shaping employment trajectories.

The observation period spans from birth to age 55, ideally the time frame during which a person who has had at least one work episode would have started working, especially considering that individuals in older cohorts often entered the labor market at a much earlier age, sometimes also at school age. The phenomena under study are modeled as simple processes, each characterized by a single episode with a single outcome. In other words, each individual can experience a non-recurring episode, and the state space of the dependent variable is binary: either the event occurs or it does not.

Event History Analysis (EHA) revolves around two central concepts: the survival function and the hazard rate. Among the tools used to analyze time-to-event data, the life table plays a key role in summarizing the empirical distribution of event occurrences over time.

The life table reports, for each time interval, the conditional probability that an individual who is still at risk at the beginning of the interval will experience the event within that specific time frame. This approach is particularly valuable when the data are grouped into discrete time intervals, offering an alternative way to estimate the survival function based on aggregated information.

The survival estimate is calculated from the conditional probabilities of failure: for interval *i, t*_*i*_ is the start time and *q*_*i*_ is the estimated conditional probability of failure. The probability of surviving to *t*_*i*_ or beyond is:


$$\hat{S}\left(t\right)=\prod_{j-1}^{i-1}\left(1-q_{j}\right)$$

where for *i* = 1 and *t*_*i*_ = 0 the survival probability is set to 1.0 (Allison, 2010).

Furthermore, we estimate the probability of event occurrence using discrete-time logistic regression models, conditioning on the fact that the individual has not yet experienced the event. The data are structured in a long format, with repeated observations for each subject across multiple time periods, continuing until the subject either experiences the event, is censored, or reaches the end of the observation window. Within this framework, the logit of the probability of experiencing the event at time *t* is modeled as a function of several covariates:


$$\begin{array}{l} \text{logit}\left(P\left(Y_{it}=1\right)\right)=\alpha +\beta_{1}{\text{SocialClass}}_{i}+\beta_{2}{\text{Education}}_{i}\\ \;+\beta_{3}{\text{Age}}_{it}+\beta_{4}{\text{Age}}_{it}^{2}+\beta_{5}{\text{Gender}}_{i}\\ \;+\beta_{6}{\text{GeographicArea}}_{i}+\beta_{7}{\text{FirstChild}}_{it}\\ \;+\beta_{8}{\text{StillInSchool}}_{it}+\beta_{9}{\text{Cohort}}_{i}\\ \;+\overset{5}{\underset{j=1}{\sum }}\overset{7}{\underset{k=1}{\sum }}\beta_{jk}\left({\text{SocialClass}}_{ij}{\text{Cohort}}_{ik}\right)\\ \;+\overset{5}{\underset{j=1}{\sum }}\overset{6}{\underset{z=1}{\sum }}\beta_{jk}\left({\text{SocialClass}}_{ij}{\text{GeographicArea}}_{iz}\right)\\ \;+\varepsilon_{it} \end{array}$$

where, *Y*_*it*_ is a binary variable indicating whether an individual experiences the event at time *t*; *SocialClass*_*i*_ refers to the individual's social class of origin, which is our focal predictor; *Education*_*it*_ measures the level of educational attained; *Age*_*it*_ and $Age_{it}^{2}$ capture non-linear age effects; *Gender*_*i*_ accounts for sex differences; *GeographicArea*_*it*_ controls for macro-regional variations in individuals' residence; *FirstChild*_*it*_ captures the effect of having had a first child; *StillInSchool*_*i*_ is an indicator for whether the individual is still enrolled in education; *SocialClass*_*ij*_ *Cohort*_*ik*_captures the interaction between social class of origin and birth cohort, resulting in a total of 35 possible combinations; *SocialClass*_*ij*_ *GeographicArea*_*ik*_captures the interaction between social class of origin and the geographical area of residence, resulting in a total of 30 possible combinations, and ε_*it*_ represents the idiosyncratic error term.

This approach allows us to estimate how these factors influence the timing of the event while accounting for time dependence and individual characteristics. Finally, gender (*Gender*_*i*_) is used as a stratification variable since the analyses will be conducted separately for males and females.

In conclusion, we aim to distinguish between the direct effects of social origin and the indirect effects mediated by the respondent's educational attainment. To disentangle these relationships, we employ the KHB decomposition method (Kohler et al., 2011), a statistical technique specifically developed to address limitations in comparing coefficients across nested nonlinear probability models, such as logistic or probit regressions. Unlike traditional approaches, the KHB method controls for rescaling bias—a distortion that arises in nonlinear models when comparing coefficients from models with and without mediators.

The KHB method decomposes the total effect of a key independent variable (in this case, social origin) into a direct effect (net of the mediator) and an indirect effect (operating through the mediator, i.e., educational attainment). This is achieved by adjusting the mediator for its association with the independent variable, and then including this adjusted component in the model. This ensures that the scale remains constant across models, allowing for an unbiased comparison of the effects before and after introducing the mediator.

In practice, we compare hazard ratios from a baseline model including social origin and controls to an extended model that also includes educational attainment. The difference between these estimates provides the indirect effect. This distinction is essential for understanding the underlying mechanisms of social stratification in early career transitions. It helps clarify whether inequalities in access to employment arise primarily from inherited advantages—such as family resources and networks—or from disparities in educational attainment. By shedding light on these pathways, our analysis provides a more nuanced perspective on how social background shapes labor market entry.

To account for the survey design and obtain unbiased estimates, survey weights were used for all the analyses described above (Pisati, 2023).

## 5 Empirical results

### 5.1 Labor market entry and its timing

**Figure 1:**
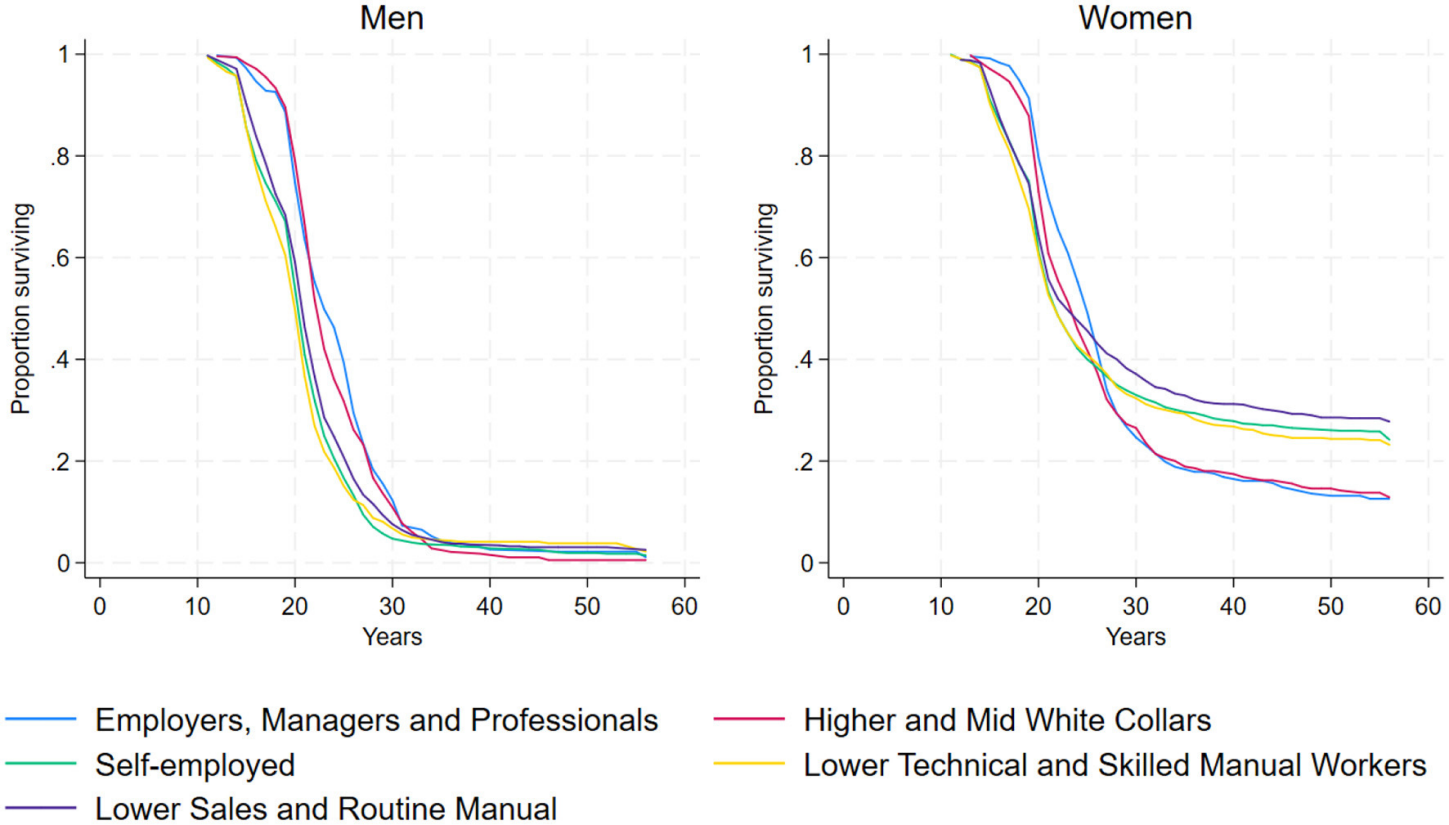
Estimated survival function by parental social class using life table method.

We begin our analysis by examining life table–based survival functions, which show the proportion of individuals who have not yet entered the labor market at each age. These estimates, presented in Figure 1 for men and women separately, provide an initial overview of how the timing of labor market entry varies by social background and gender.

For men, the survival functions indicate that individuals from Higher and Mid-White Collars and Employers, Managers and Professionals backgrounds experience a delayed entry into the labor market until around the age of 30, at which point the survival functions begin to overlap. For women a clear distinction emerges between higher and lower social backgrounds, with women from Employer and Higher and Mid-White Collar families experiencing a delayed entry into the labor market until around the age of 25. Interestingly, after that point, the proportion of women remaining out of the labor market and belonging to the two highest classes continues to decline, while the proportion of women from the most disadvantaged backgrounds who do not enter the labor market remains above 20%.

These patterns are further illustrated in Tables 1, 2, which report the 25th, median, and 75th percentiles of survival time by social class. Among men, earlier entry is observed in the lower-status groups—especially the self-employed and lower technical workers—with median entry ages between 19 and 20 years. Among higher-status men, the median rises to 22–23 years.

**Table 1:** Median age of entry into the labor market for men.

**Social class**	**Survival time**		
	**25%**	**Median**	**75%**
Employers, managers and professionals	19–20	22–23	26–27
Higher and mid white collars	19–20	21–22	26–27
Self-employed	16–17	19–20	22–23
Lower technical and skilled manual workers	15–16	19–20	22–23
Lower sales and routine manual	17–18	20–21	23–24
Total	17–18	20–21	24–25

**Table 2:** Median age of entry into the labor market for women.

**Social class**	**Survival time**		
	**25%**	**Median**	**75%**
Employers, managers and professionals	20–21	24–25	29–30
Higher and mid white collars	19–20	22–23	30–31
Self-employed	18–19	21–22	**54–55**
Lower technical and skilled manual workers	17–18	21–22	44–45
Lower sales and routine manual	18–19	22–23	**54–55**
Total	18–19	22–23	**54–55**

Values in bold indicate subgroups with small sample sizes within the dataset.

This pattern suggests that men from lower-status enter the labor market earlier, likely due to financial constraints or limited access to higher education. Individuals with a self-employed parent often enter the labor market more quickly as they can leverage parental businesses, benefiting from existing resources, networks, and forms of economic and cultural capital passed down through the family (Schizzerotto, 2002). Children of self-employed workers tend to enter the labor market more quickly because their parents can create tailor-made job opportunities for them. This reflects a well-documented outcome (Cobalti and Schizzerotto, 1994) of the intergenerational reproduction strategies typically employed by this social class.

For women, the pattern differs. The median age of labor market entry is later for women (22–23 years) than for men (20–21 years), with earliest transitions observed among self-employed and lower technical backgrounds (21–22 years), and latest among employers (24–25 years). This suggests that while lower-status men tend to enter the labor market early out of necessity, women from higher-status backgrounds may experience more structured education-to-work transitions that facilitate labor market entry. Conversely, the lower incidence rate among women from routine manual backgrounds may indicate barriers to employment, possibly due to lower education levels, fewer professional networks, or gendered labor market expectations in certain industries.

Furthermore, gender differences are reflected in the variation of survival times as indicated by the interquartile range (IQR). Among men, the IQRs across social classes typically span 6–8 years, suggesting relatively concentrated transitions into the labor market. In contrast, women exhibit much wider IQRs, especially among lower-status groups. This substantial variation highlights that women's labor market entry is not only delayed relative to men, but also far more heterogeneous—particularly among the most disadvantaged, for whom stable employment may be postponed or never achieved.

### 5.2 Social background and labor market entry: regression findings

The results from the regression analysis (Tables 3, 4) confirm that social origin significantly influences labor market entry. Compared to individuals from higher-status backgrounds (employers, managers, and professionals), those from lower-status social origins generally enter the labor market earlier.

**Table 3:** Average Marginal Effect (AME) for men.

	**AME**	**SE**	***p*-value**	**95 % CI**
**SOCIAL CLASS**				
**Employers, managers and professionals (reference)**				
Higher and mid white collars	0.010	0.003	0.00	0.003–0.016
Self-employed	0.036	0.004	0.00	0.028–0.045
Lower technical and skilled manual workers	0.045	0.005	0.00	0.035–0.055
Lower sales and routine manual	0.036	0.005	0.00	0.027–0.045
**COHORT**				
**1926–1945 (silent generation) (reference)**				
1946–1955 (Baby boom 1)	−0.026	0.010	0.01	−0.046 to −0.006
1956–1965 (Baby boom 2)	−0.048	0.010	0.00	−0.068 to −0.028
1966–1980 (Generation X)	−0.052	0.010	0.00	−0.072 to −0.033
1981–1995 (Generation Y)	−0.051	0.010	0.00	−0.070 to −0.032
1996–2004 (Generation Z)	−0.052	0.012	0.00	−0.076 to −0.027
**EDUCATION**				
**No school (reference)**				
Primary	0.006	0.003	0.05	0.000–0.012
Lower secondary	0.025	0.005	0.00	0.015–0.034
Upper secondary	0.076	0.011	0.00	0.053–0.099
Tertiary	0.205	0.025	0.00	0.157–0.254
Post-tertiary	0.381	0.096	0.00	0.192–0.572
**GEOGRAPHIC AREA**				
**Northwest (reference)**				
Northeast	−0.009	0.006	0.16	−0.022 to 0.004
Central	−0.021	0.005	0.00	−0.031 to −0.011
South	−0.028	0.005	0.00	−0.038 to −0.019
Islands	−0.013	0.005	0.02	−0.024 to −0.002
Foreign Countries	−0.037	0.007	0.00	−0.051 to −0.023
**FIRST CHILD**				
**No child (reference)**				
At least one child	0.008	0.010	0.42	−0.012 to 0.028
**STILL IN SCHOOL**				
**No (reference)**				
Yes	−0.045	0.004	0.00	−0.053 to −0.037

**Table 4:** Average Marginal Effect (AME) for women.

	**AME**	**SE**	***p*-value**	**95 % CI**
**SOCIAL CLASS**				
**Employers, managers and professionals (reference)**				
Higher and mid white collars	0.003	0.002	0.05	0.000–0.006
Self-employed	0.015	0.002	0.00	0.012–0.019
Lower technical and skilled manual workers	0.021	0.002	0.00	0.016–0.025
Lower sales and routine manual	0.019	0.002	0.00	0.015–0.023
**COHORT**				
**1926–1945 (silent generation) (reference)**				
1946–1955 (Baby boom 1)	−0.003	0.003	0.46	−0.011 to 0.005
1956–1965 (Baby boom 2)	−0.018	0.003	0.00	−0.026 to −0.010
1966–1980 (Generation X)	−0.020	0.003	0.00	−0.028 to −0.013
1981–1995 (Generation Y)	−0.023	0.003	0.00	−0.031 to −0.016
1996-2004 (Generation Z)	−0.027	0.003	0.00	−0.036 to −0.018
**EDUCATION**				
**No school (reference)**				
Primary	0.006	0.001	0.00	0.004–0.008
Lower Secondary	0.023	0.002	0.00	0.020–0.026
Upper Secondary	0.100	0.007	0.00	0.087–0.113
Tertiary	0.278	0.020	0.00	0.239–0.316
Post-tertiary	0.470	0.043	0.00	0.386–0.554
**GEOGRAPHIC AREA**				
**Northwest (Reference)**				
Northeast	−0.0005	0.003	0.88	−0.007 to 0.006
Central	−0.018	0.003	0.00	−0.024 to −0.011
South	−0.033	0.002	0.00	−0.038 to −0.028
Islands	−0.027	0.003	0.00	−0.032 to −0.022
Foreign Countries	−0.032	0.003	0.00	−0.038 to −0.026
**FIRST CHILD**				
**No child (Reference)**				
At least one child	−0.010	0.002	0.00	−0.014 to −0.006
**STILL IN SCHOOL**				
**No (Reference)**				
Yes	−0.022	0.002	0.00	−0.026 to −0.017

Among men, those who belong to the lower technical and skilled manual workers class are 4.2 percentage points more likely to enter employment than those from employer backgrounds (*p* < 0.001), with slightly weaker effects for those from self-employed and lower sales and routine manual backgrounds (+3.4 p.p., *p* < 0.001). A similar, though weaker, trend is observed for women, where coming from a self-employed family increases labor market entry by 1.5 percentage points (*p* < 0.001), while those from lower-skilled manual and routine backgrounds see an increase between 1.9 and 2.1 percentage points (*p* < 0.001).

Beyond social origin, individual educational attainment is a key determinant in labor market entry processes, acting both as a compensatory mechanism and as an amplifier of inequalities linked to socioeconomic background. Higher levels of education are significantly associated with an increased probability of transitioning into employment for both men and women. However, the positive effect of education appears to be particularly pronounced for women, suggesting that they may require higher educational credentials to achieve labor market entry rates comparable to those of men. Men with a tertiary education degree exhibit a 19.7 percentage point higher probability of entering the labor market compared to those without a primary school diploma. Among women, this effect is even more pronounced, reaching 27.8 percentage points, highlighting the differential role of education in shaping employment entry trajectories across gender.

The relationship between age and labor market entry is non-linear, with employment probabilities peaking in early adulthood before declining. Specifically, the maximum of the function, controlling for the other variables, (i.e., the age associated with the highest probability of entering into the labor market) is 24 years for men and 12 years for women.

This pattern is further compounded by generational shifts, as younger cohorts face increasing delays in workforce entry. Compared to older generations, Generation X, Generation Y, and the Generation Z exhibit significantly lower employment probabilities, reflecting rising job precarity, and shifting entry requirements that have made labor market integration progressively more challenging.

Finally, our results show that labor market entry is strongly shaped by geography, with respondents from Southern Italy, the Islands, and abroad facing lower probabilities of employment, reflecting territorial inequalities linked to structural labor market differences, job scarcity, and weaker professional networks. These macro-regional disparities are more pronounced for women, who also face additional barriers related to family conditions. For men residing in the South, the entry risk rate is 2.7 percentage points lower compared to those in the North-West. Among women, this reduction is even more pronounced, reaching 3.3 percentage points, highlighting stronger regional disparities in labor market entry.

While parenthood does not significantly affect men's employment entry, it reduces women's probability of securing a job, potentially reinforcing traditional gender roles and constraining their labor market integration.

These findings confirm H1, showing that individuals from lower social backgrounds enter the labor market earlier, while those from higher-status families tend to delay entry, likely due to extended education and greater financial support. However, this raises an important question: to what extent does education mediate the influence of social background?

### 5.3 The mediating role of education

While social origin clearly shapes labor market entry, it remains unclear whether these disparities stem primarily from educational pathways or if social background exerts an independent influence beyond education. To disentangle these mechanisms, we employ KHB decomposition analysis, which allows us to assess the direct effect of social origin vs. the indirect effect mediated by education.

The decomposition results (Tables 5, 6) reveal that education does not fully account for the effect of social background. Instead of reducing differences across social origins, controlling for one's own educational attainment amplifies them, as the Direct Effects (after accounting for education) are larger than the Total Effects (before accounting for education). This suggests that education was masking rather than mediating the true effect of social origin, revealing a stronger direct impact of social class on labor market entry once differences in schooling are considered.

**Table 5:** Total, Direct, and Indirect effect of class of origin on labor market entry for men.

	**Coefficient**	**SE**	***p*-value**	**95 % CI**
**EMPLOYERS, MANAGERS AND PROFESSIONALS (REFERENCE)**				
**Higher and mid white collars**				
Total effect	0.24	0.10	0.02	0.05–0.43
Direct effect	0.27	0.10	0.01	0.08–0.46
Indirect effect	−0.03	0.02	0.11	−0.07 to 0.007
**Self-employed**				
Total effect	0.69	0.08	0.00	0.52–0.85
Direct effect	0.81	0.09	0.00	0.65–0.98
Indirect effect	−0.13	0.02	0.00	−0.17 to −0.09
**Lower technical and skilled manual workers**				
Total effect	0.80	0.09	0.00	0.62–0.97
Direct effect	0.95	0.09	0.00	0.78–1.13
Indirect effect	−0.16	0.02	0.00	−0.20 to −0.11
**Lower sales and routine manual**				
Total effect	0.69	0.09	0.00	0.50–0.84
Direct effect	0.81	0.09	0.00	0.64–0.98
Indirect effect	−0.14	0.02	0.00	−0.18 to −0.09

**Table 6:** Total, direct, and indirect effect of class of origin on labor market entry for women.

	**Coefficient**	**SE**	***p*-value**	**95 % CI**
**EMPLOYERS, MANAGERS AND PROFESSIONALS (REFERENCE)**				
**Higher and mid white collars**				
Total effect	0.16	0.09	0.08	−0.02 to 0.35
Direct effect	0.17	0.09	0.06	−0.01 to 0.36
Indirect effect	−0.01	0.03	0.74	−0.06 to 0.04
**Self-employed**				
Total effect	0.24	0.08	0.00	0.08–0.40
Direct effect	0.66	0.08	0.00	0.50–0.82
Indirect effect	−0.43	0.03	0.00	−0.49 to −0.37
**Lower technical and skilled manual workers**				
Total effect	0.42	0.09	0.00	0.25–0.59
Direct effect	0.82	0.09	0.00	0.65–0.99
Indirect effect	−0.40	0.03	0.00	−0.46 to −0.34
**Lower sales and routine manual**				
Total effect	0.26	0.08	0.00	0.10–0.43
Direct effect	0.76	0.09	0.00	0.60–0.93
Indirect effect	−0.50	0.03	0.00	−0.56 to −0.44

**Figure 2:**
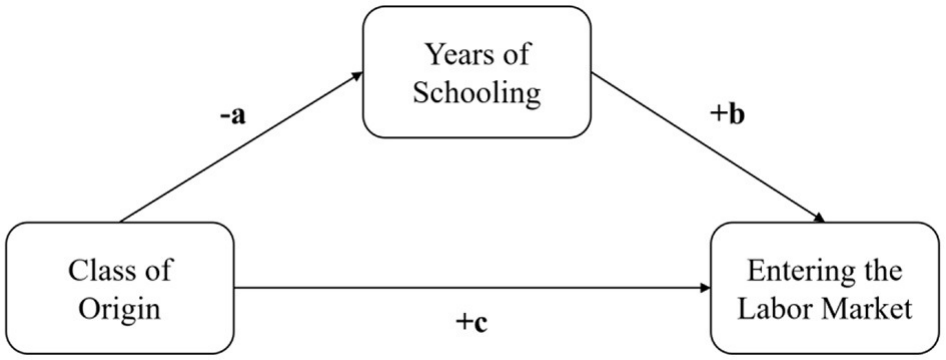
The single-mediator model.

This pattern corresponds to what is known in the literature as 'inconsistent mediation' (MacKinnon et al., 2000, 2007), where the mediated effect through educational attainment has the opposite sign of the direct effect of social class on the risk of entering the labor market, thereby weakening the total effect of social class once years of schooling are accounted for (Figure 2). Specifically, *a* is the effect of class origin on the years spent in school, *b* is the effect of the years of schooling on the probability of entering the labor market and, finally, *c* is the direct effect of class of origin on the probability of entering the labor market. The parameter *a* is negative, individuals coming from any given class compared with offsprings from the highest class spend less years in school. The other two parameters are positive as shown in the model estimates: studying more allows individuals to enter the labor market earlier, and women and men from lower classes are more likely to enter the labor market.

For men, the decomposition shows that those from lower-status social backgrounds (e.g., skilled manual, routine manual, and self-employed families) enter the labor market earlier than those from employer backgrounds. However, controlling for education makes these differences even larger, with the effect of social background increasing rather than decreasing. This pattern suggests that education was previously absorbing part of the influence of social background, likely because individuals from lower-status families tend to have lower educational attainment, which initially made the total effect appear smaller. Once education is accounted for, the remaining direct effect of social origin is stronger, pointing to additional mechanisms—such as informal job networks, access to early employment opportunities, or employer biases—that reinforce these differences independently of education.

For women, the same trend emerges but is even more pronounced. Women from lower-skilled and self-employed backgrounds are more likely to enter employment earlier than those from employer backgrounds. However, once education is controlled for, these differences increase substantially, indicating that education was suppressing the full effect of social background. This means that while women from lower-status backgrounds may obtain higher education, this does not fully offset their disadvantage in labor market entry. Other factors—such as gendered hiring practices, occupational segregation, or weaker professional networks—likely play a critical role in shaping their employment transitions.

Overall, these findings challenge the idea that education alone equalizes labor market opportunities. Instead, they confirm H2, demonstrating that educational attainment mediates the relationship between social origin and labor market entry, as individuals from higher-status backgrounds tend to delay entry due to prolonged education. However, education does not fully eliminate class-based disparities—controlling for educational attainment amplifies rather than erases the effect of social origin, indicating that other mechanisms, such as social capital, financial resources, and labor market networks, still play a role. These patterns are particularly pronounced for women, aligning with H3: the mediating role of education is stronger for women, as they require higher qualifications to enter the labor market at rates comparable to men. This suggests that while education facilitates labor market integration, it does not fully mitigate class—and gender-based inequalities in employment transitions.

### 5.4 Cohort and regional differences in labor market entry

The findings from the decomposition analysis confirm that social origin continues to exert a strong influence on labor market entry, even after accounting for educational attainment. This invites further exploration of how this influence has evolved across successive birth cohorts, in light of broader structural transformations affecting youth transitions. Moreover, given the marked regional differences in labor market conditions and institutional support across Italy, it is important to consider how class-based disparities in employment entry vary by geographical area.

**Figure 3:**
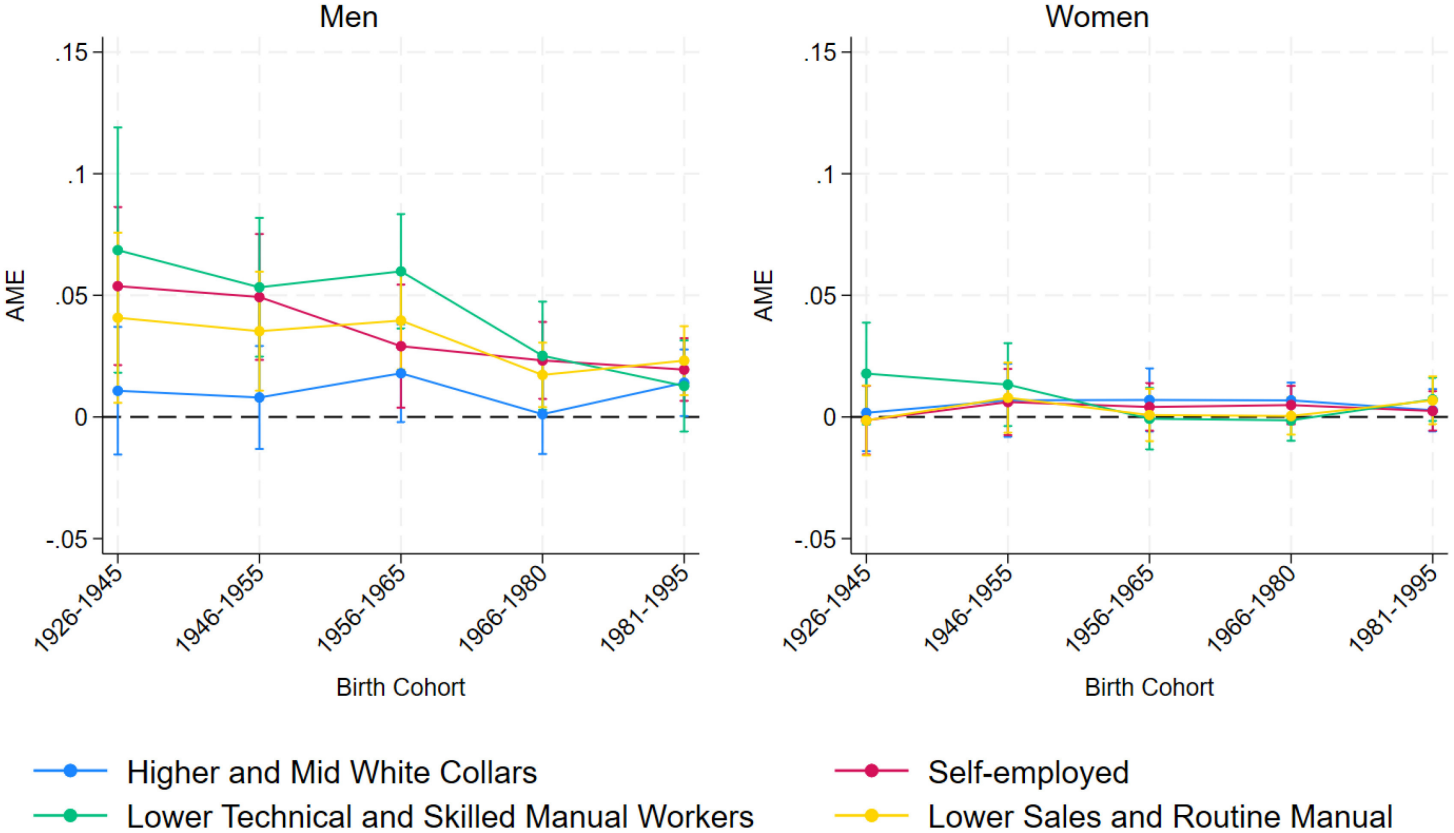
Average Marginal Effects (AME) of parental social class on the probability of entering the labor market by birth cohort.

Specifically, if social class disparities in labor market entry lose significance across successive cohorts, this lends support to postmodernist theories. To test this hypothesis (H4), we incorporated interaction terms between social class of origin and birth cohorts into our models. Figure 3 illustrates the average marginal effect of each social class on the probability of labor market entry across different birth cohorts.

Specifically, for men, individuals from lower-status social backgrounds (Self-employed, Lower Technical and Skilled Manual Workers, and Lower Sales and Routine Manual) generally enter the labor market earlier than those from Employers, Managers, and Professionals. This pattern remains relatively stable over time, with some fluctuations across cohorts. If anything, there is evidence of a partial reduction in the gap between the two highest-status classes and the other three, suggesting slight convergence in market entry timing. Among women, the trend is different: the effect of each class displayed in the plot is not statistically different from that of the highest one across cohorts. Therefore, H4 is partially supported, with social class disparities in market entry showing a somewhat convergent trend for male but relative stability for female respondents.

**Figure 4:**
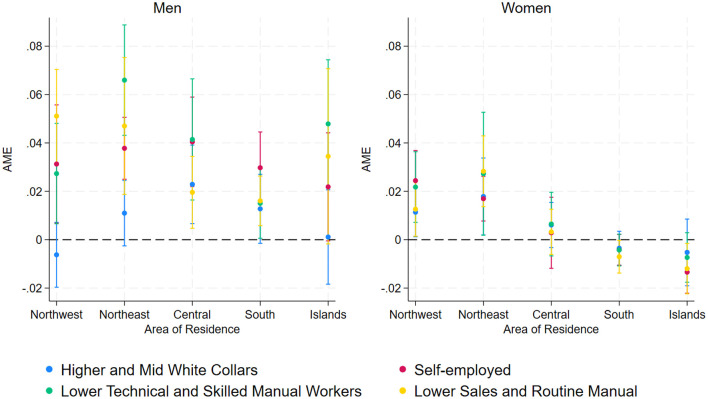
Average Marginal Effects (AME) of parental social class on the probability of entering the labor market by geographical area.

Finally, Figure 4 shows the average marginal effect of each social class on the probability of labor market entry across different geographical areas. Among men, even sons from the Higher and Mid White Collars class enter the labor market earlier than those from the Employers, Managers, and Professionals class if they live in the northeast and central Italy. Among women, regional disparities are more evident, with lower-status individuals in the northwest and northeast showing a higher probability of labor market entry compared to daughters from the Employers, Managers, and Professionals class. However, in the south and islands, class effects are weaker, with little distinction between social backgrounds, likely reflecting broader constraints in labor market access that affect all groups more uniformly. Instead, class effects are more pronounced in the wealthier northern regions, while in the south and islands, weaker differentiation suggests that broader labor market constraints limit opportunities for all groups.

## 6 Discussion

This study has examined the role of social class of origin in shaping labor market entry in Italy, with a particular focus on the mediating effect of education. Using retrospective longitudinal data from the ITA.LI survey and event history analysis techniques, our findings confirm that social background significantly influences the timing of employment entry, though its effects vary by gender, birth cohort, and area of residence.

Our results support the persistence of social class inequalities in employment transitions, reinforcing theories that emphasize the continued influence of family background on life course trajectories (Bourdieu, 1984; Elder, 1985). Specifically, we find that individuals from lower socioeconomic backgrounds tend to enter the labor market earlier, often due to financial constraints and limited access to extended education. This is consistent with arguments that highlight the role of economic, cultural, and social resources in shaping transition-to-adulthood patterns, as individuals from disadvantaged backgrounds are more likely to prioritize immediate labor market entry over prolonged education (Billari et al., 2019). Conversely, individuals from higher-status families, particularly those with professional or managerial backgrounds, tend to delay the transition to first employment, leveraging longer educational trajectories to secure better career opportunities. This finding aligns with research emphasizing how education serves as a mechanism of social stratification, allowing privileged groups to maintain their advantages (McLanahan, 2004; Furstenberg, 2010).

However, while education can serve as a critical mediator in employment transitions, our results suggest that it does not fully neutralize the effects of social origin on labor market entry. Instead, our decomposition analysis highlights that controlling for education amplifies rather than diminishes social class disparities, pointing to additional mechanisms such as social capital, network advantages, and employer preferences that perpetuate inequalities beyond educational attainment. This interpretation is supported by resource-based perspectives (Bourdieu, 1984; Granovetter, 1973) which highlight that economic, cultural, and social capital within the family of origin continues to shape life course trajectories. In Italy, where personal connections and informal recruitment channels play an important role in labor market entry, family networks and inherited social capital can provide advantages that extend beyond formal education credentials.

Our results also highlight significant gender differences persisting in labor market entry patterns. While men generally transition into employment earlier, women face longer delays, particularly those from higher-status backgrounds. In contrast, women from lower social origins enter the labor market sooner, often in lower-skilled and less secure positions, reinforcing structural disadvantages. These findings underscore the interplay between gender, social class, and employment trajectories, highlighting persistent inequalities in occupational opportunities.

This pattern aligns with earlier findings in the literature that emphasize how women's labor market pathways are shaped by both class-based stratification and gendered institutional constraints (Blau and Kahn, 2017; Naldini and Saraceno, 2022). In Italy's familistic welfare context, women—especially those from less privileged backgrounds—often face pressure to enter the labor market early, albeit into less secure roles (Mencarini and Solera, 2011). Meanwhile, women from higher-status families, despite attaining longer educational trajectories, still experience a slower or more uncertain transition into employment, not necessarily due to caregiving responsibilities or the lack of childcare services at the point of entry, but rather due to the continued devaluation of women's work and limited early career opportunities (Bozzon, 2008).

Our decomposition analysis further confirms that education has a stronger mediating role for women—consistent with prior studies showing that women need higher credentials to access comparable positions (Blau and Kahn, 2017). Yet, this advantage is constrained: once education is accounted for, the residual effect of social origin remains significant, suggesting that neither educational attainment nor qualifications alone are sufficient to overcome class- and gender-based inequalities in labor market transitions.

The study reveals that while younger cohorts experience delays in labor market entry due to rising educational participation, economic transformations, and labor market deregulation, class-based disparities have remained largely stable over time. This aligns with arguments suggesting that while educational expansion and labor market transformations have altered employment trajectories, they have not necessarily eliminated class-based inequalities (Goldthorpe, 2000; Piketty, 2014). Among men, a slight convergence is observed, but individuals from lower-status backgrounds continue to enter the labor market earlier than those from the highest-status group, consistent with research highlighting the enduring role of financial constraints and the necessity of early employment for lower-class youth (Billari et al., 2019). Among women, class differences are more complex. As shown by Mencarini and Solera (2011), women's employment trajectories are generally more fragmented and more influenced by sector and contract type than by social origin alone. Our findings echo this, showing weaker class effects for women, particularly in the later cohorts. However, disadvantaged women continue to face higher risks of early labor market entry into unstable roles, indicating persistent vertical and horizontal segmentation.

Regional variations shape labor market entry dynamics, but class-based disparities are not necessarily stronger in Southern Italy. Among women, class effects are more pronounced in the wealthier northern regions, where lower-status individuals exhibit higher employment probabilities compared to their higher-status counterparts. In contrast, in the south and islands, class differences are less distinct, suggesting broader labor market constraints limit opportunities for all groups. Among men, class effects appear relatively stable across regions, with no strong evidence that disparities are systematically larger in the north or south. This aligns with prior research on Italy's territorial divide, where structural labor market differences and economic conditions shape employment transitions in complex ways (Reyneri, 1997; Pugliese and Rebeggiani, 1997).

Despite its contributions, this study has certain limitations. First, while our analysis accounts for key sociodemographic variables, unobserved factors such as motivation, individual agency, and informal job networks may also play a role in shaping employment transitions. Additionally, our data primarily focus on labor market entry rather than career progression, limiting insights into long-term occupational mobility. Future research should explore how early labor market experiences translate into career outcomes and whether initial disadvantages persist or are mitigated over time.

Overall, our findings confirm that social origin remains a significant determinant of labor market entry in Italy, despite changes in education and labor market structures. While increased access to education has contributed to greater opportunities, it has not entirely eliminated class-based disparities in employment transitions. Given the persistent influence of social origin on young people's entry into the Italian labor market, despite expanded educational access, policy interventions should focus on improving the school-to-work transition through targeted support for disadvantaged groups. Efforts should include strengthening vocational and career guidance services in secondary and tertiary education, promoting equitable access to high-quality internships and apprenticeships, and investing in public employment services to bridge the gap between education and labor demand. Additionally, addressing the unequal distribution of social capital by supporting mentorship programs and expanding professional networks for youth from lower socioeconomic backgrounds could help mitigate inherited disadvantages. Finally, regional disparities highlight the need for place-based strategies that enhance labor market opportunities in southern and peripheral areas, particularly for young women, who face compounded barriers related to class and gender.

Addressing these persistent inequalities requires targeted policies aimed at improving school-to-work linkages, expanding access to quality education, and fostering equitable labor market conditions that enable individuals from diverse backgrounds to navigate employment transitions more effectively.

## Data Availability

The data analyzed in this study is subject to licenses/restrictions and will be made public in January 2026. Requests to access these datasets should be directed to mario.lucchini@unimib.it.
